# Quantitative proteomics analysis of *Angiostrongylus vasorum*-induced alterations in dog serum sheds light on the pathogenesis of canine angiostrongylosis

**DOI:** 10.1038/s41598-020-79459-9

**Published:** 2021-01-11

**Authors:** Lucienne Tritten, Nina Gillis-Germitsch, Tobias Kockmann, Manuela Schnyder

**Affiliations:** 1 Institute of Parasitology, Vetsuisse Faculty, University of Zurich, Zurich, Switzerland; 2 Graduate School for Cellular and Biomedical Sciences, University of Bern, Bern, Switzerland; 3 Functional Genomics Center Zurich, ETH/UZH Zurich, Zurich, Switzerland

**Keywords:** Parasitic infection, Mass spectrometry, Proteomic analysis, Infection, Molecular biology

## Abstract

Blood contains hundreds of proteins, reflecting ongoing cellular processes and immune reactions. Infections with the blood-dwelling cardiopulmonary nematode *Angiostrongylus vasorum* in dogs manifest with a broad spectrum of clinical signs including respiratory distress, bleeding diathesis and neurological signs, and are associated with a perturbed blood protein profile in dogs. However, current knowledge does not completely explain the observed pathologies induced by *A. vasorum* infections, including bleeding disorders. Using sera from experimentally infected dogs, dog serum proteome was analysed by quantitative mass spectrometry methods over several time points before and after inoculation. Following computational analysis, we identified 139 up- and downregulated proteins after infection (log2 ratio cut-off ≥ 1.0; q-value ≤ 0.05). Among upregulated proteins were chitinase 3-like 1 and pulmonary surfactant-associated protein B (log2 fold-changes ≥ 5). Pathway enrichment revealed the complement (especially the lectin pathway) and coagulation cascades as significantly affected upon analysis of downregulated proteins. Among them were mannan-binding lectin serine peptidases, ficolin, and coagulation factor XIII-B. These results bring new elements towards understanding the underlying pathomechanisms of bleeding diatheses observed in some *A. vasorum*-infected dogs.

## Introduction

*Angiostrongylus vasorum* is a metastrongylid cardiopulmonary nematode parasite of dogs and wild carnivores such as red foxes, causing a range of clinical signs with potentially fatal outcomes in dogs. After ingestion of infected gastropods acting as intermediate hosts and a 6 weeks prepatent period, adult worms reside in the pulmonary arteries and the right side of the heart of their definitive hosts^1–3^. There, they release numerous eggs. Hatched first stage larvae migrate through the lungs, inducing verminous pneumonia. If left untreated, the infection is associated with a high fatality rate in dogs. Respiratory disease, bleeding disorders, and other diverse and unspecific signs may result from an infection with *A. vasorum*^4,5^. Dogs showing coagulation deficiency are more prone to die as a result of the infection^6–8^. The heterogeneous clinical picture of canine angiostrongylosis hampers efficient diagnosis as well as timely and appropriate treatment. The pathogenesis of the diverse array of clinical signs and especially coagulopathies associated with the infection remain poorly understood. Hypocoagulability is more frequently observed than hypercoagulability^6,9^ and approximately 20–30% of cases were estimated to present with bleeding tendencies or neurological signs induced by bleeding^4,10^. Multiple hypotheses have been formulated to explain the onset of coagulation disorders; the issue remains nonetheless unresolved to date. Antigenic stimulation causing chronic disseminated intravascular coagulation (DIC), acquired deficits in von Willebrand and other coagulation factors, (immune-mediated) thrombocytopenia (however, usually transient)^11–14^, thrombocytopathy^7^, immune complex-mediated stimulation of coagulation, and the secretion of anticoagulants by the parasite have been proposed^6,11–16^. Up to 43% of infected dogs (with and without visible clinical signs of bleeding) showed hyperfibrinolysis^7^.

Analysing the blood proteome may reveal valuable information: it contains hundreds of proteins, reflecting dynamically ongoing cellular processes and immune reactions. In addition, *A. vasorum* resides in blood, and blood-dwelling parasites are known to modify their hosts’ haemostatic system^17^. Several studies reported a perturbed protein profile in *A. vasorum*-infected dogs. Some described high levels of total protein, especially of the globulin fraction^18,19^. Others noted a transient increase in α1- or α2-globulins and β-globulin fractions in infected dogs^15,20^. One study reported β-globulins to decrease on day 20 post-infection with *A. vasorum* before a significant increase by day 45, compared to control animals^20^. However, these reports do not provide a thorough and systematic analysis of all protein fractions.

The analysis of a dysregulated serum protein profile may therefore guide us towards a better understanding of the molecular biology behind pathogenesis and be useful in the quest for diagnostic and prognostic biomarkers. A mass-spectrometric approach enables the deep analysis of the dynamics and specificity of a tissue’s proteome in response to a health condition and may point at molecular events compromising the maintenance of homeostasis (e.g.^21–23^).

This report represents a first in-depth analysis of the dynamics of the dog serum proteome over three to five time points over the course of an experimental infection with *A. vasorum*. Our study suggests that the complement system, the lectin pathway in particular, as well as the innate immune system are negatively impacted by the infection progressing to a chronic phase. In addition, coagulation factor XIII, subunit B, involved in the stabilization of fibrin clots, appeared to be depleted. These novel insights warrant further targeted investigations, since they provide leads towards an explanation for the development of *A. vasorum*-driven coagulopathies.

## Results

**Figure 1:**
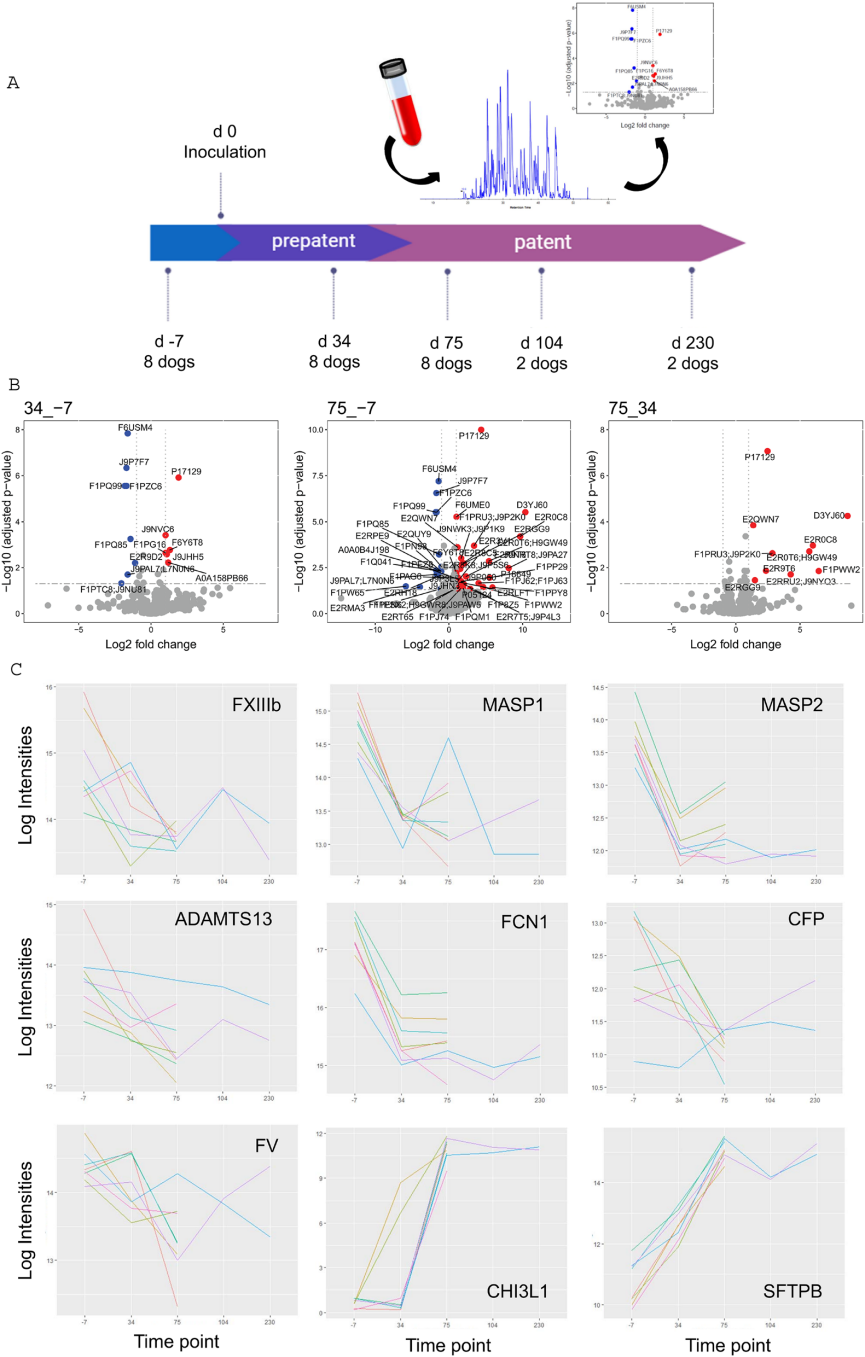
Study design and analysis of protein expression across time and individuals. (**A**) Serum sampling schedule and workflow. Blood was drawn at several time points before and after infection with *A. vasorum*, across the prepatent and patent phases. Samples were processed and subjected to LC–MS proteomic analysis to identify alterations in protein levels over time; (**B**) Volcano plots representing pairwise comparisons across the first three time points. 1: comparison day 34 to day-7, 2: comparison day 75 to day 34, 3: comparison day 75 to day-7. Each protein is represented by one dot and labelled with a Uniprot accession. Significant proteins are defined by a combination of adjusted *p*-value ≤ 0.05, and log2 fold-change ≥ 1 (upregulated; red) or ≤ -1 (downregulated; blue); (**C**) Profile plots for selected proteins across time. The x-axis represents time points, log_2_ protein intensities are shown in the y-axis. *ADAMTS13* ADAM metallopeptidase with thrombospondin type 1 motif 13 (J9P430), *CFP* complement factor properdin (E2RH18), *FXIII-B* coagulation factor XIII, polypeptide B (F1Q041), *FV* coagulation factor V (F1PN98), *FCN1* ficolin (collagen/fibrinogen containing lectin) 1 (J9P7F7), *MASP1* mannan-binding lectin serine peptidase 1 (F1PQ85), *MASP2* mannan-binding lectin serine peptidase 2 (F6USM4), *CHI3L1* chitinase-3-like protein 1 precursor (D3YJ60), *SFTPB* pulmonary surfactant protein B (P17129). Time points: day-7, 34, 75, 104, and 230 p.i. (**B**,**C**) images were produced with R^62,63^.

Serum from eight dogs was sampled at three time points: (i) before infection, (ii) during pre-patency, and (iii) one to three time points after patency (Fig. 1a), subjected to reduction of the protein dynamic concentration range by applying combinatorial peptide ligand libraries, and screened for differential abundant proteins by data independent acquisition (DIA) executed on a liquid chromatography coupled to mass spectrometry (LC–MS) system. A list of identified proteins and peptides is provided in Table S1. The number of proteins that was found significantly up- or downregulated (adjusted *p*-value ≤ 0.05) across the different pairwise comparisons ranged between 0 and 49 (Table S1).

### Serum profile changes from day-7 to day 75

Several protein expression changes were observed at day 34 p.i. already (Fig. 1b): 8 proteins appeared significantly downregulated; 6 were upregulated. Pulmonary surfactant-associated protein B (SFTPB; P17129) further accumulated in dog sera and another ten proteins appeared newly up-regulated (for a total of 11 up-regulated proteins) at day 75 p.i. compared to day 34, with no down-regulated protein (Fig. 1b). Alteration of protein expression appeared most pronounced at day 75 p.i., compared to before infection (Fig. 1b), where the levels of a total of 34 proteins were increased and 15 were decreased (listed in Supplementary Table S1). Levels of chitinase 3-like protein (CHI3L1, D3YJ60), SFTPB (E2R0C8), and a saposin B-type domain-containing protein (J9NRT8/J9PA27) increased the most, with fold-changes up to 20 times between days 75 and -7. Comparing the same time points, the greatest fold-changes for downregulated proteins were observed for fibrinogen beta chain (FGB; F1PW65) and secreted protein acidic and cysteine rich (SPARC; E2RMA3), although just below the *p*-value cut-off (set at 0.05). Most significantly down-regulated, this time according to the adjusted *p*-values, were mannan binding lectin serine peptidases 1 and 2 (MASP1 and -2; F1PQ99, F6USM4), and ficolin-1 (FCN1; J9P7F7).

### Downregulated proteins over time

**Figure 2:**
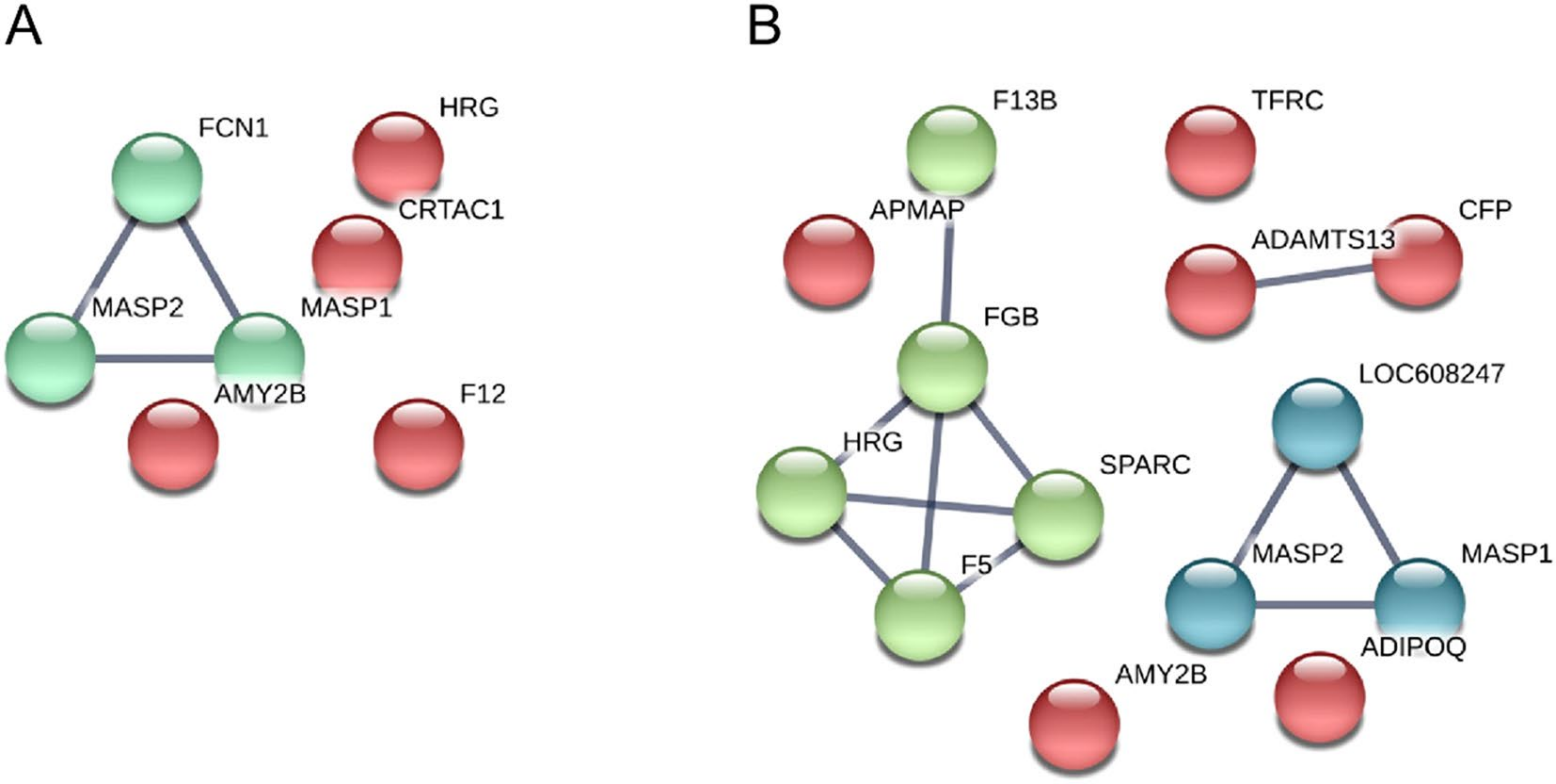
Functional protein–protein interactions among downregulated proteins. (**A**) downregulated proteins at day 34 compared to day-7, (**B**) downregulated proteins at day 75 compared to day-7. *ADAMTS13* ADAM metallopeptidase with thrombospondin type 1 motif 13, *ADIPOQ* adiponectin precursor, *AMY2B* amylase α2B, *APMAP* adipocyte plasma membrane associated protein, *CFP* complement factor properdin, *CRTAC1* cartilage acidic protein 1, *F12* coagulation factor XII, *F13B* coagulation factor XIII, polypeptide B, *F5* coagulation factor V, *FCN1/LOC608247* ficolin (collagen/fibrinogen containing lectin) 1, *FGB* fibrinogen β chain, *HRG* histidine-rich glycoprotein, *MASP1* mannan-binding lectin serine peptidase 1, *MASP2* mannan-binding lectin serine peptidase 2, *SPARC* secreted protein acidic, cysteine-rich, *TRFC* transferrin receptor protein 1.

Functional protein–protein interactions for downregulated proteins at day 34 and at day 75 p.i. compared to day-7, respectively, are depicted in Fig. 2. MASP1, MASP2, and FCN1 (and LOC608247, a dog orthologue of FCN1) are known interactors. Their levels are consistently decreased at both days 34 and 75 p.i. compared to before infection; however, no further decrease was observed from day 34 to 75. At day 75, two further interaction networks appeared. The first is composed of coagulation factors FXIII-B and FV; the latter interacting in turn with a histidine-rich glycoprotein (HRG; F1PZC6) and SPARC.

The most common GO terms (resulting from Blast2GO search) associated with downregulated proteins both at days 34 and 75 compared to day-7 were “complement activation, lectin pathway” (GO:0001867) and “proteolysis” (GO:0006508). Similarly, the most common molecular functions were “calcium ion binding” (GO:0005509) and “serine-type endopeptidase activity” (GO:0004252), at both time points.

STRING analysis of significantly downregulated proteins at day 34 compared to day-7 revealed an overrepresentation of the Reactome pathway “ficolins bind to repetitive carbohydrate structures of the target cell surface” (CFA-2855086, FDR = 1.13e−08), due to the presence of MASP1, MASP2, and FCN1. MASP1, MASP2, and F12 (E2R9D2) have trypsin activities (Pfam domain, FDR = 9.26e−05), and together with cartilage acidic protein 1 (CRTAC1; F1PTC8), they share an “EGF-like domain” (FDR = 7.06e−06).

At day 75, MASP1, MASP2, and FCN1 contributed to the enrichment of “ficolins bind to repetitive carbohydrate structures of the target cell surface” (FDR = 2.26e−07), to “initial triggering of complement” (CFA-166663, FDR = 2.26e−07), and “complement cascade” (CFA-166658, FDR = 9.27e−07), together with properdin (E2RH18). FV (F1PN98), FXIII-B (F1Q041) and FGB contributed to “common pathway of fibrin clot formation” (CFA-140875, FDR = 2.03e−06). FV, FGB, SPARC, and HRG are associated with the “platelet degranulation” pathway (CFA-114608, FDR = 6.22e−06). FV, FXIII-B, FGB, SPARC, and HRG contributed to “haemostasis” (CFA-109582, FDR = 8.54e−05). Sushi domains (MASP1, MASP2, and FXIII-B) and CUB domains (MASP1, MASP2, and ADAMTS13 (E2QUY9)) were overrepresented among the dataset as well.

Changes appeared generally consistent across the 8 individual dogs. Several examples are depicted in Fig. 1c.

### Upregulated proteins over time

Among the 6 upregulated proteins at day 34 compared to day-7, one item (A0A158PB66), was recognized as an *A. cantonensis* uncharacterized protein.

SFTPB (P17129 in Fig. 1b) displayed a 3.8-fold and an 8.7-fold increase at days 34 and 75 compared to day-7, respectively (see Supplementary Fig. S1). A known interactor, napsin A (NAPSA; F1PWW2), was also found to be upregulated at this time point. Reactome pathways “scavenging of haem from plasma” (CFA-216880), “cell surface interactions at the vascular wall” (CFA-202733) and “haemostasis” (CFA-109582) were found enriched (FDR ≤ 0.05) based on the presence of two dog proteins with antigen binding functions (J9NVC6 and J9JHH5; not shown). Reactome pathways “innate immune system” (CFA-168249, FDR = 3.09e−08) and “immune system” (CFA-168256, FDR = 1.32e−05) were overrepresented among upregulated proteins (13 out of 34 proteins in total) at day 75 compared to before infection (Supplementary Fig S1). Several immunoglobulin-like domains as well as saposin-like domains were enriched as well (Pfam domains, FDRs ≤ 0.001). CHI3L1, strongly upregulated at day 75 (20-fold), interacts with interleukin enhancer binding factor 2 (ILF2; E2R9T6), also upregulated. Six out of 34 upregulated proteins resulting from the same time point comparison were described as ‘Ig-like domain-containing proteins’.

### Trends for dysregulated candidates at later time points

For two dogs, sera sampled on days 104 and 230 p.i. were analysed additionally. Given the lack of sufficient replicates, these two late time points only serve the purpose of showing trends. All five time points are shown in Fig. 1c for 9 selected candidates that were significantly up- or downregulated by day 75. There was no difference in protein expression between days 75 and 104, or between days 104 and 230 (summarized in Supplementary Table S1). The only difference between expression profiles at days 230 and 75 was a reduced expression in thrombospondin 4 (THBS4; F1PSS2) at the later time point (not shown). Proteins like SFTPB appeared consistently upregulated at days 104 and 230 p.i. compared to day-7, as well as between days 230 and 34. Similarly, CHI3L1 remained present at higher levels at the two later time points compared to days-7 and 34. MASP1 and -2, as well as HRG and FCN1 were consistently present at lower levels at days 104 and 230 compared to day-7, as was the case at days 34 and 75. THBS4 was depleted at day 230 compared to all the three first time points.

Generally, protein abundance (log2 intensities) measured at days 104 and 230, when the infection can be called chronic, confirm the trends observed in earlier patent infections (day 75). Although no conclusion can be drawn from only 2 dogs, it seems that the baseline protein levels (from day-7) are not recovered at those later time points.

### Confirmation of differential protein abundance by targeted mass spectrometry

**Figure 3:**
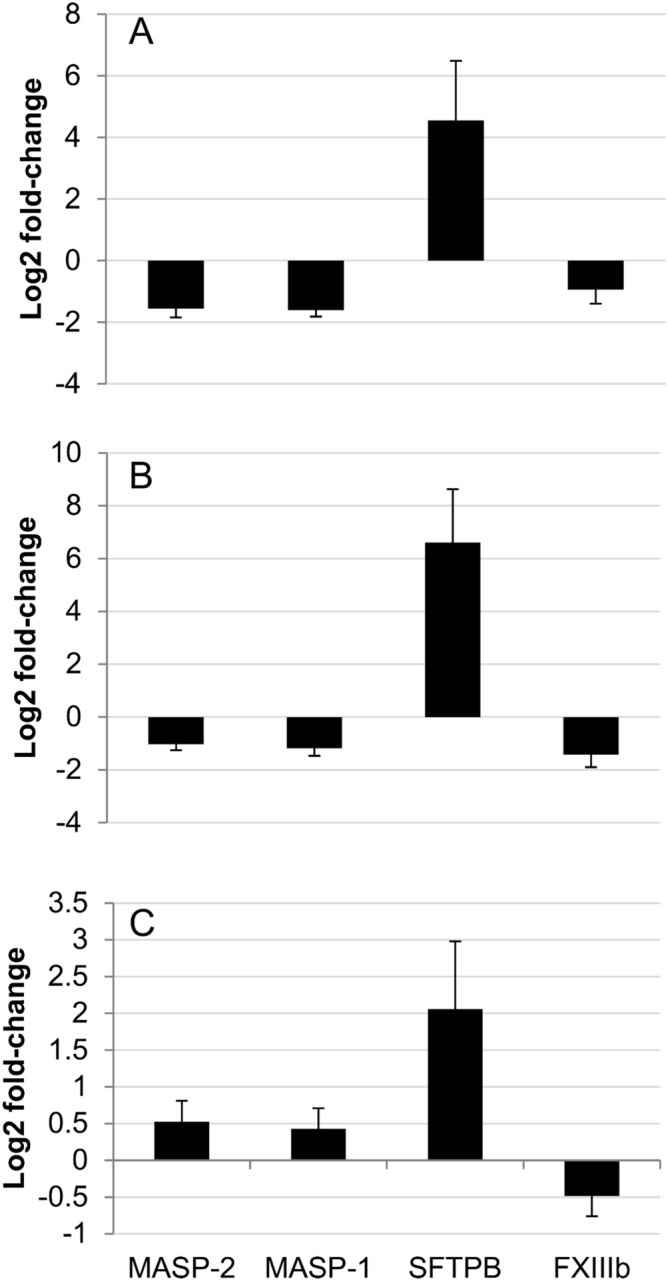
Average protein expression for 4 candidates across time points in PRM assay. Graphs depict log2 fold-changes of measured protein intensities, standard errors are indicated. (**A**) comparison day 34/day-7; (**B**) comparison day 75/day-7; (**C**) comparison day 75/day 34. Three proteins were found to be downregulated over the course of the infection in the main study (DIA experiments and analysis in Spectronaut and MSstat): MASP-1 and MASP-2 (F1PQ85 and F6USM4), and FXIII-B (F1Q041). The last protein, SFTPB (P17129), was upregulated over time in the main study.

Due to lacking immunoreagents we developed targeted LC–MS assays for relative quantification of SFTPB, FXIII-B, MASP-1, and MASP-2 (Table S2). In summary, targeted re-quantification of the above described serum samples indeed in PRM (Parallel Reaction Monitoring) assays confirmed the fold-changes estimated by DIA in expression between days-7 and 75 (Fig. 3): SFTPB (P17129) Log2 FC = 6.6 ± 2.0 (log2 FC = 5.5 ± 1.3 in the main study), FXIII-B (F1Q041) log2 FC = − 1.4 ± 0.5 (log2 FC = − 1.1 ± 0.2 in the main study), MASP-2 (F6USM4) log2 FC = − 1.0 ± 0.2 (log2 FC = − 1.4 ± 0.1 in the main study), MASP-1 (F1PQ85) log2 FC = − 1.2 ± 0.3 (log2 FC  = − 1.3 ± 0.2 in the main study).

## Discussion

The rationale behind this study was to gain knowledge about infection-related serum proteomic patterns, which in turn may explain the development of coagulation disorders and potential further pathogenic aspects in *A. vasorum*-infected dogs. To our knowledge, this is the first deep proteomic analysis of blood from infected animals covering all infection phases. In order to validate the protein level variations across samples and time points, we successfully developed PRM assays including synthetic reference peptides, the first of its kind for dog serum proteins.

Alterations of the serum protein profile appeared generally consistent across the 8 individual dogs up to day 75 p.i. and data from two dogs at days 104 and 230 suggest that these alterations tend to persist. We emphasize that data from these two later time points have an indicative function, rather than conclusive, due to the number of donor individuals available being reduced to only two. Our study dogs did not show visible signs of bleeding upon medical checks (unpublished results, and^5,24^). Despite the lack of haematological data for the study dogs at the examined time points, our experience shows that laboratory *A. vasorum* infections lead to severe pulmonary changes including vascular thrombosis, but only mild haematological changes, without overt signs of bleeding over such a short period of time^5,25^. If the measured coagulation times were essentially within reference ranges throughout the experiments, the activated partial thromboplastin time (aPTT) was significantly higher in dogs receiving a higher infective load at days 63 and 70 p.i. A mild to moderate thrombocytopenia was observed in patent infections, especially when the worm burden was high^5^. Other studies of natural and laboratory infections reported further changes at the level of coagulation factor activity and coagulability^6,7^. Here, we discuss a few elements of particular interest in this context.

Among upregulated proteins, our data reflect the ongoing anti-helminth immune response and the damage inflicted to the infected lungs. CHI3L1 is a secretory glycoprotein expressed by different cell types. Chitinase-like proteins (CLP) are enzymatically inactive molecules^27^. Each mammalian species exhibits a different collection of CLP-encoding genes^28^. In mice, all CLPs including CHI3L1 are commonly upregulated in the context of T-helper 2-driven inflammation of the lungs. In the helminth model *Nippostrongylus brasiliensis*, CLPs were shown to exert antiparasitic functions through IL-17-mediated recruitment of neutrophils, a reaction that causes lung damage^27^. A known interactor of CHI3L1, ILF2, was also found to be upregulated in the current study. According to Reactome, they contribute to the exocytosis of specific granule lumen proteins by neutrophils, and so, play roles in phagocytosis and killing extracellular pathogens^29^. ILF2 is an essential transcription factor for T cell expression of the IL-2 gene.

SFTPB is a highly hydrophobic apoprotein of the family of c-type lectins. Upon the inspiratory stretch of the alveolar cell layer, alveolar pneumocytes secrete surfactant-containing lamellar bodies, which are then reorganized into the highly surface-active tubular myelin and large multilamellar vesicles^30^. In human patients with acute respiratory distress syndrome (ARDS), plasma levels of surfactant protein B are elevated as a result of tissue insult^31^. Similarly, the lungs of *A. vasorum*-infected dogs are constantly injured by hatching first-stage larvae. Like other saposin-like proteins (e.g. NK-lysin, granulysin, and amoebapore), SFTPB exhibits antimicrobial activity by interacting with lipid components of cell membranes. Human surfactant protein B was shown to induce bacterial killing through aggregation and membrane permeabilization^32^. Bacterial aggregation caused by other surfactant proteins was proposed to enhance phagocytosis and favour complement activation among other roles^33^. However, the membranolytic activity is not selective for bacteria, as red blood cell lysis also occurs in vitro. Because its effects are inhibited by phospholipids, to which it is naturally associated in vivo, the role of endogenous surfactant protein B in alveolar host defence may be restricted^32^. However, others found that the predominant forms of alveolar immunoreactive surfactant protein B, proprotein and processing intermediate, are not bound to surfactant lipids, which would facilitate entry into the circulation^31^, leaving the possibility for host defence properties outside the lungs as well.

Our data indicate that several players of the complement and coagulation pathways are expressed at lower levels with the infection progressing to a chronic stage (day 75 p.i.), as compared to before infection. Evolutionarily related enzymatic cascades contributing to host defence, coagulation and complement cascades are closely interwoven with each other and show many interactions. For example, platelets may become activated as a result of complement activation, and vice versa^34^. Or, complement C5a also increases the expression of tissue factor on leukocytes^35^.

Reactome pathways “ficolins bind to repetitive carbohydrate structures of the target cell surface”, “initial triggering of complement”, “complement cascade”, “common pathway of fibrin clot formation, and “haemostasis” were overrepresented in our list of downregulated candidates. The complement system plays a crucial role in the innate defence against a wide array of pathogens, including helminths^36^. Its activation leads to a series of proteolytic events that ultimately result in opsonisation and lysis of the pathogen, as well as the generation of inflammation. The complement system is nowadays also regarded as an important link between the innate and adaptive immune responses. It can be activated by three different routes: the classical, lectin, or alternative pathways^37^. The lectin pathway occupies a particular place at an important crossroad between complement and coagulation. It gets activated by microbial sugars and relies on pattern recognition receptors (PRRs; in the form of e.g., mannose-binding lectins or ficolins, which recognize pathogen-associated molecular patterns (PAMPs)). PRRs form complexes with MASP1/MASP2. Binding of PRRs on a pathogen’s surface leads to the activation of MASPs, which cleave C2 and C4, enabling the formation of C3 convertase (the converging point of all three complement activation pathways). The initiation of the complement cascade may also depend on other proteins. For example, properdin (CFP; also down-regulated at day 75) is capable of activating the alternative complement pathway by stabilizing the C3 convertase and by promoting its de novo assembly.

FCN1 is a lectin component of secretory granules, primarily expressed in granulocytes and monocytes. FCN1 may contribute to the activation of the complement system via the lectin pathway^38^. Since it binds back to granulocytes upon release, human FCN1 plasma levels are relatively low but reflect granulocyte counts^39^. In addition, plasma concentrations vary across time as a result of the lectin pathway activation/consumption^38^.

MASPs are homologs of C1r and C1s of the classical complement pathway. MASP-2 is necessary for mannose-binding lectin (MBL)-mediated complement activation via the lectin pathway, while the functional role of MASP-1 in the MBL complex is unclear^40^.

MASP-1 and MASP-2 were among the most strongly downregulated proteins at day 75. These proteases participate in the complement activation via the lectin pathway. Indeed, both are able to cleave the coagulation factors prothrombin, fibrinogen, factor XIII, and thrombin-activatable fibrinolysis inhibitor in vitro^41,42^. Thus, MASPs may contribute to the formation and stabilization of cross-linked fibrin. In vitro, MASP-1 and MASP-2 are triggered by the combined effect of platelet activation and fibrin formation^43^. MBL/MASP-1/2 complex has thrombin-like activity^40^. In line with this, mice lacking MBL or MASP-1 demonstrate a significantly prolonged bleeding time^40^. Altogether, these results show that different key components of the coagulation cascade, as well as several activation pathways of the complement cascade are found at decreased levels after 75 days p.i. with *A. vasorum* in dogs. Therefore, lacking amounts of these proteins may explain (at least partially) the onset of bleeding disorders; however, further targeted studies will be required in order to validate this hypothesis.

Coagulation factors V and XIII-B were detected at lower levels at day 75 compared to before infection. Together with fibrinogen β chain among other molecules, they are key players of fibrin clot formation. Factor V is the plasma cofactor for the prothrombinase complex that activates prothrombin to thrombin. It is both present in platelet alpha-granules and in plasma; low FV levels are seen in liver or platelet disorders^44^. Overall, human patients with lower FV levels are more likely to have bleeding episodes than those with higher levels; the severity of the clinical picture is determined by the FV activity level^44^. In *A. vasorum* infections, decreased FV activity has been described in both experimentally and naturally infected animals^13,14,26^.

FXIII is the last zymogen to become activated in the coagulation cascades and stabilizes fibrin clots. This circulating heterotetrameric pro-transglutaminase complex is composed of two catalytic FXIII-A and two protective/regulatory FXIII-B subunits^45^. The roles of FXIII-B remain incompletely resolved; it bears 10 sushi domains (or Complement Control Protein modules) and resembles complement factor H based on sequence and structural homology^46^. FXIII deficiency may be acquired, as a result of immune-mediated inhibition, defective synthesis and/or consumption of either of FXIII subunits^47^. In human blood, potential interactors of FXIII-B include fibrinogen-α, -β, and -γ chains, complement C1q, and C3. In addition, alpha-2-macroglobulin was suggested to be a direct interactor of FXIII-B^45^. Alpha-2-macroglobulin is a known substrate for FXIIIa, although no clear functional role has been discovered in this context; to date there are no reports of its direct interaction with FXIII-B^45,48^. Alpha-2-macroglobulin can inhibit both coagulation and fibrinolysis by acting on thrombin or plasmin^49^. Hence, it was proposed that the interaction of alpha-2-macroglobulin with FXIII or FXIII-B subunit might fine-tune the two different processes^45^. The association between FXIII-B and fibrinogen was shown several times; the subunit playing important roles in the formation of a stable fibrin clot. Thus, FXIII-B deficiency may result in increased bleeding tendency through impaired fibrin stabilization due to decreased FXIIIa activation by thrombin and secondary FXIII-B deficiency arising from enhanced circulatory clearance^46^. FXIII’s association with the complement system has been described, whereby FXIII-mediated covalent cross-linking of fibrin to complement C3 could contribute inflammatory roles in pro-thrombotic states^50^.

ADAMTS13, another downregulated protein, is a von Willebrand factor-cleaving protease showing several thrombospondin domains; DIC has been associated with reduced ADAMTS13 activity^51^. HRG interacts with a large panel of ligands and acts as a regulator of coagulation and fibrinolysis as both anti- and pro-fibrinolytic activities have been proposed^52^. In septic mice, where DIC is part of the clinical picture, HRG levels were significantly decreased. HRG supplementation antagonized the pro-DIC state, the immunothrombotic events, and inhibited lung inflammation^53^.

Several hypotheses have been formulated underlying behind the pathogenesis of coagulopathies in *A. vasorum* infected dogs^7^. DIC and consumption coagulopathy have been the most popular explanations, sometimes combined with other above-mentioned features^13,14,16,26^. DIC is a life-threatening condition to both humans and animals; it is associated with poor prognosis in dogs. It may arise as a secondary complication in numerous disorders and is characterized by the systemic (and excessive) activation of coagulation leading to the formation of microthromboses. In humans, multiple organ failure and simultaneous bleeding consequently occur due to consumption of platelets, clotting factors, and fibrinogen^6,7,54^.

Under DIC conditions, the fibrinolytic activity is largely suppressed in experimental models. A pro-fibrinolytic response is almost immediately followed by a suppression of fibrinolysis due to a sustained increase in plasma levels of plasminogen activator inhibitor, type 1^54^. None of these elements appeared dysregulated in our *A. vasorum*-infected dog sera.

Substantial differences exist between controlled laboratory infections and dogs presented to the clinics: the number of ingested infectious larvae, the duration of the infection, and the occurrence of repeated infections are usually unknown in naturally infected animals. The health status, age, as well as genetic variation across breeds may also impact the outcome of an infection. Our study relies on serum and not on plasma; hence, lesser amounts of some clotting agents, i.e. fibrinogen, factor II, V, and VIII^55^, are expected to be seen as a result of the induced clot formation. Due to the applied column-based protein enrichment, upregulation of proteins such as albumin, usually present at high levels in serum even at a healthy baseline level, was not expected.

## Conclusion

Our data do not support the claim of classic DIC to occur. Instead, we showed that the coagulation and complement cascades are affected over the course of the infection, the lectin pathway in particular. After summarizing potential reasons for the onset of bleeding in *A. vasorum* infections, Sigrist and colleagues wrote “A different, previously undetected pathomechanism leading to bleeding diathesis in *A. vasorum* infection therefore is expected”^7^. Several coagulation constituents being present at reduced levels in serum from dogs harbouring a patent infection, we propose a consumption of some key factors to be potentially at the origin of the onset of bleeding disorders. Concomitantly, upregulated components (i.e. CHI3L1 and SFTP) of the immune system support the important role of triggered immunological reactions contributing to the clinical picture of canine angiostrongylosis. Findings from this work do not provide a conclusive and mechanistic explanation for the coagulopathies in *A. vasorum* infections but provide a few leads to shape future research on the role of FXIII-B (for its roles in fibrin stabilization), MASP-1 and -2 (for their thrombin-like activity and as regulators of fibrin clot formation and stabilization), ADAMTS13 (involved in von Willebrand factor cleavage), and HRG (as a regulator of coagulation and fibrinolysis) in particular.

## Methods

### Ethics statement

Ethical clearance for the initial studies conducted in 2008^24,25^ was obtained from the Cantonal Veterinary Office of Zurich (permission number 185/2008). All experiments were conducted in accordance with the Swiss cantonal and national regulations on animal experimentation.

### Experimental design

Briefly, eight beagles (5 males and 3 females, 11 months of age at the start of the experiment) were inoculated with 200 *A. vasorum* third-stage larvae as described in previous papers based on the same animals^24,25^. Each animal was subjected to a weekly clinical check-up with a focus on respiratory abnormalities, as well as faecal examination to monitor the infection through the excretion of first-stage larvae. There was no systematic haematological analysis. Each dog was sampled repeatedly over the course of the infection. Time points were selected in order to reflect serum profiles (i) before infection (one week prior to infection; day-7), (ii) during prepatency (day 34 post-infection (p.i.)), and (iii) after patency (day 75 p.i.; see Fig. 1a). Hence, each dog is represented by an uninfected (baseline) sample and at least two time points during ongoing infection. Serum from two further time points (days 104 and 230 p.i.) was analysed additionally. Due to requirements associated with animal experimentation, samples from late time points were available for two dogs only.

### Serum collection and processing

Blood was collected from the jugular vein into serum tubes (Greiner Bio-One Vacuette Z serum clot activator (silica particles)) and spun down for 10 min at 1800 × *g*, prior to storage at − 20 °C for 9 years. Protein enrichment was performed using the ProteoMiner small capacity kit (Bio-Rad) following the manufacturer’s instructions. Proteins were precipitated using trichloroacetic acid (TCA) according to a modified protocol by Thermo Fisher/Pierce. Briefly, 50 µl protein eluates were mixed with 450 µl dH_2_O, 100 µl sodium deoxycholate 0.15% (w/v), and 100 µl TCA 72% (w/v). After a 30 min incubation at 4 °C samples were vortexed, spun 30 min at 16,900 × *g*, 4 °C, and pellets were washed 3 times with pure ice-cold acetone. Pellets were air-dried and resuspended in 50 µl 4% SDS, Tris–HCl 0.1 M, pH = 7.6, 0.1 M DTT. Protein in samples was quantified by Qubit protein assay (Thermo Fisher Scientific). Thirty µg protein were further alkylated and digested using a modified filter-aided sample preparation^56^ (see Supplementary Methods). Dried peptides were resuspended in 3% acetonitrile (ACN), 0.1% formic acid (FA) and frozen at − 20 °C pending use.

### Data-independent acquisition and analysis

Samples were processed in randomized blocks, and analysed in random order. A total of 28 label-free analyses were performed in a LC–MS/MS experiment using a data-independent acquisition (DIA) protocol. Quality check consisted of an assessment of trends in precursor m/z errors, and fragment ion errors, as well as of the proportion of missed cleavage and consistency in cysteine modification across samples. Peptide concentration was adjusted to 0.5 µg/ml in 3% ACN, 0.1% FA, and retention time normalization peptides (iRT, Biognosys) were added at a 1:20 ratio. Three μl purified peptide mixture (1.5 µg on column) per sample was analysed in random order in DIA mode on a hybrid quadrupole-Orbitrap mass spectrometer (Q Exactive HF, Thermo Scientific) operated in line with a nano UHPLC system (Easy-nLC 1000, Thermo Scientific). For further details see Supplementary Methods.

Spectral libraries (data-dependent acquisition mode) were produced for each time point based on pooled samples from each dog in equal amounts. Raw data was processed in Proteome Discoverer (v. 2.1; Thermo Fisher Scientific) and Spectronaut (v. 11; Biognosys), using the reference proteomes of *Canis lupus familiaris* (UP000002254), *A. costaricensis* and *A. cantonensis* (UP000050601, UP000035642) from UniProt (accessed on 28th of August 2017). Peptide-level data was further exported from Spectronaut and analysed using the MSStats R package (v 3.12.3) for statistical relative quantification and significance analysis^57^ and the following parameters: log transformation: 2; normalization method: equalizeMedians, summary method: Tukey’s median polish; censored values in intensity column: null; MBimpute: false. Note: The fitted model took repeated sampling from the same study subject (dog) into account. Significance cut-offs for up- or downregulation were defined by an adjusted *p*-value ≤ 0.05 and fold-changes ≥ 2, or ≤ − 2. Pathway enrichment was done using STRING DB v11. Up- or downregulated proteins, as defined by the cutoffs set in MSstats, were submitted to the STRING online tool^58^. Networks were produced by linking interacting proteins based on confidence in the interaction. Functional protein–protein interactions were built based on the highest confidence score (≥ 0.9), computed as in^59^. STRING pathway enrichment relies on serval databases (Biocarta, BioCyc, GO, KEGG, and Reactome). Gene ontology terms associated with sets of proteins were obtained using Blast2GO v 4.1^60^. The mass spectrometry proteomics data have been deposited to the ProteomeXchange Consortium via the PRIDE^61^ partner repository with the dataset identifier PXD016881. For further details see Supplementary Methods.

### Confirmation of differential protein abundance by targeted mass spectrometry

Details on PRM assay development, characterization, and application to serum samples can be found in Supplementary Methods.

## Supplementary Information

Supplementary Information 1.

Supplementary Information 2.

Supplementary Information 3.

Supplementary Information 4.

Supplementary Information 5.

## Data Availability

The mass spectrometry proteomics data have been deposited to the ProteomeXchange Consortium via the PRIDE^61^ partner repository with the dataset identifier PXD016881 and PXD016887. Targeted proteomics data was submitted to PanoramaPublic and can be accessed using [https://panoramaweb.org/whmPyd.url]. The R code (MSstats) is available upon request to the corresponding authors.
