# Supplementary material for: Quantitative proteomics analysis of Angiostrongylus vasorum-induced alterations in dog serum sheds light on the pathogenesis of canine angiostrongylosis
Source: Sci Rep. 2021 Jan 11;11:283. doi: 10.1038/s41598-020-79459-9 (PMC7801463; doi:10.1038/s41598-020-79459-9)
Supplement: Supplementary file 1 — Supplementary Information 1. [file 41598_2020_79459_MOESM1_ESM.docx]

## Supplementary Methods

### Filter-aided sample preparation

Thirty µg protein were mixed with 200 µl 8 M urea in 100 mM Tris-HCl pH 8.2, prior to be loaded onto Microcon 30 filters (Millipore, Cat. MRCF0R030) and spun at 14,000 x *g* for 25 min at 35 °C. Samples were washed again with 200 µl 8 M urea buffer and 100 µl 0.05 M iodoacetamide were added to the filter units, as described ^1^. Samples were shaken for 1 min in a thermo-mixer (600 rpm), incubated 5 min at room temperature (RT) and spun as before. Filters were washed 2 x with 100 µl 8 M urea buffer and with 100 µl 0.5 M NaCl. Finally, 120 µl 0.05 M Triethylammoniumbicarbonate and 1.5 µl sequencing grade modified trypsin 0.4 µg/µl (Promega, V5113) were added, mixed 1 min (600 rpm) and incubated overnight in a wet cell at RT. Filter units were spun at 14,000 x g for 25 min and the flow-through acidified with trifluoroacetic acid (TFA) to reach a 0.5 % final concentration. Peptides were then desalted using C18 stage tips ^2^. Briefly, equilibrated columns were loaded with 150 µl sample (in 3 % acetonitrile (ACN), 0.1 % TFA) and spun at 2,000 x g for 1 min. Samples were washed 2 x with 150 µl 3 % ACN, 0.1 % TFA and eluted in 150 µl 60 % ACN, 0.1 % TFA. Samples were placed into a speed-vac and dried to completeness.

### Data-independent acquisition (DIA)

For each sample 3 μl of purified peptide mixture was loaded onto a fused-silica column (150 mm x 75 um ID) filled with porous reverse-phase chromatography resin (Reprosil-Pur C18 AQ, 1.9 μm spherical silica particles, 120 Å pore diameter, M/N: r119.aq, Dr. Maisch GmbH, Ammerbuch, Germany). Bound peptides were eluted from the analytical column by running a linear gradient from 5 % to 35 % B in 120 min (Solvent A: 0.1% FA in water, solvent B: 0.1% FA in acetonitrile) at a flow rate of 300 nl/min. Eluted peptides were injected into the MS utilizing a nano ESI source (10 μm fused-silica spray emitter and Digital PicoView 565, O/N: DPV-550-565, New Objective, Woburn, MA). MS1 scans covering 350-1800 m/z were recorded in profile mode at a resolution (R) of 60,000 (at 200 m/z) using positive polarity and automated gain control (target value: 3e6; maximum injection time (maxIT): 200 ms). Each MS1 scan was followed by 35 multiplexed fragment ion scans (DIA scans) covering the m/z range of 400 to 1100 in 20 m/z windows. DIA scans were recorded at a R = 30’000 in centroid mode using an AGC target of 1e6 and maxIT of 55 ms. Isolated precursors were fragmented using higher energy collisionally activated dissociation (HCD) at a normalized collision energy (NCE) of 28. Fixed first mass was set to 100 m/z.

Database searches were performed in Proteome Discoverer software (v. 2.1, Thermo Fisher Scientific), using SequestHT as search engine ^3^, which was applied to search the raw data against the Uniprot fasta databases for *Canis lupus familiaris* (UP000002254), *A. costaricensis* and *A. cantonensis* (UP000050601, UP000035642) accessed on Aug 28, 2017, along with the Biognosys iRT peptides sequence. A maximum of two missed cleavages were allowed. Carbamidomethylation of cysteine was chosen as fixed modification, while oxidation of methionine and N-terminal acetylation of proteins, methionine loss, methionine loss plus acetylation were selected as dynamic modifications. False discovery rate (FDR) was set to 1 % for peptide spectrum matches and proteins based on Percolator results ^4^. Spectronaut (v. 11; Biognosys) was applied for the label-free quantification of proteins with factory settings, including the generation of spectral libraries. The same sequence databases and modification parameters were used as for the generation of spectral libraries. The FDR was set at 1 % for both peptide spectrum matches and proteins.

### PRM assay development and characterization

For protein quantification by Parallel Reaction Monitoring (PRM) four proteins of interest (F1Q041, P17129, F1PQ85, and F6USM4) were selected from differential expression analysis results. Proteins for validation were selected on the base of their important roles in the coagulation and complement cascades. Peptides were selected based on their proteotypic profiles, (short) length, and XIC MS2 alignments across samples. For each target protein 2 to 3 proteotypic peptides were synthetized, ranging from 8 to 24 amino acids in length (C-terminal isotope label at Arginine (+10 Da) or Lysine (+8 Da), carbamidomethyl Cysteine, PEPotec grade 2, Thermo Scientific, listed in Supplementary Table S2). Quality of SIL peptide synthesis was validated by HR/AM LC-MS analysis in DDA mode on a Q Exactive HF (Thermo Scientific) operated in line with a nanoAcquity M-class (Waters). The M-class was operated in single-pump trapping 75-um scale configuration having nanoEase M/Z Symmetry C18 100A, 5 um, 1/PK 180 µm x 20 mm Trap column (Waters Part No. 186008821) and nanoEase M/Z HSS C18 T3 Col 100 A, 1.8 µm, 1/PK 75 µm X 250 mm (Waters Part No. 186008818) columns installed. Resulting MS2 spectra were searched against the UniProt reference proteome UP000002254 (dog) using the Mascot search engine. Annotated spectra were used to create an iRT-calibrated spectral library applying the BiblioSpec spectral library tools in Skyline (v. 4.2 ^5^). In addition, a predicted spectral library was generated using Prosit ^6^ (NCE = 27, z = 2). Both libraries have been deposited in PanoramaPublic. According to the spectral libraries, the top 5 fragment ions from the y series were selected for PRM recording (excluding y1, y2 and ions around the precursor). An initial unscheduled PRM run (data not shown) targeting the SIL peptides indicated insufficient signal response for five peptides. These peptides were therefore excluded from being used for targeted quantification. For the remaining peptides calibration curves were established using the procedure suggested by the CPTAC assay characterization guidelines [<https://proteomics.cancer.gov/assay-portal/about/assay-characterization-guidance-documents>]. In short, a six point serial dilution series (5-fold dilution steps ranging from ~17 nM to ~54 μM) of SIL peptides and one blank sample in 0.5 μg/ml digested dog serum matrix were prepared. Three μl of each sample were injected in technical duplicates using unscheduled PRM on the same LC-MS system as indicated above. Peptides were separated by a linear gradient from 5 to 35% B in 40 min at a flow rate of 300 nl/min (A: water, 0.1% FA; B: acetonitrile, 0.1% FA). Precursors were quadrupole isolated using a 1.4 m/z window. PRM scans were executed in centroid mode at a R = 120’00 using AGC (target value: 1e5, maxIT: 247 ms) and applying HCD fragmentation at a NCE = 27. The resulting data was analysed in Skyline. No misscleavage was allowed. Carbamidomethylation of cysteine and oxidation of methionine were allowed. In brief, for each peptide a linear model was fitted explaining the measured peak area (signal) as a function of theoretical SIL peptide amount. In addition, the figures of merit LOD and LOQ were determined. Details can be found in supplementary figures (Fig. S2).

### Targeted protein quantification by PRM

The 28 precipitated dog serum samples, which were previously screened by DIA, were again digested and cleaned as described above. SIL peptides were spiked into digested serum samples at a constant concentration close to the endogenous counterpart (actual concentrations can be found in Supplementary Table S2) and measured by scheduled PRM on a nanoAcquity M-class (Waters) directly coupled to a Q Exactive HF-X (Thermo). The M-class was operated in single-pump trapping 75-um scale configuration having nanoEase M/Z Symmetry C18 100A, 5 um, 1/PK 180 µm x 20 mm Trap column (Part No. 186008821) and Waters nanoEase M/Z HSS C18 T3 Col 100 A, 1.8 µm, 1/PK 75 µm X 250 mm (Part No. 186008818) columns installed. Peptides were separated by a linear gradient from 5 to 35% B in 40 min at a flow rate of 300 nl/min (A: water, 0.1% FA; B: acetonitrile, 0.1% FA). Precursors were quadrupole isolated using a 1.4 m/z window +/- 5 min around the expected retention time. PRM scans were executed in centroid mode at a R = 120’00 using AGC (target value: 1e5, maxIT: 247 ms) and applying HCD fragmentation at a NCE = 27. Resulting scan data was analysed in Skyline using SIL peptide signals (heavy) for single point calibration of endogenous signals (light). One of 9 SIL peptides had to be excluded for being present below limit of detection. In addition, four samples (two samples from day -7, one from day 34, and one from day 75) were completely excluded due to distorted total ion chromatograms (TIC), likely caused by incomplete digestion. The light signals of the remaining 24 samples were normalized according to TIC area and used for relative quantification on a log2 scale. Effect size (fold change) and significance estimates (p-values) are reported as output by the MSstats quantification module in Skyline.

## References

1. Wiśniewski, J. R., Zougman, A., Nagaraj, N. & Mann, M. Universal sample preparation method for proteome analysis. *Nat. Methods* **6**, 359–362 (2009).

2. Rappsilber, J., Ishihama, Y. & Mann, M. Stop and Go Extraction Tips for Matrix-Assisted Laser Desorption/Ionization, Nanoelectrospray, and LC/MS Sample Pretreatment in Proteomics. *Anal. Chem.* **75**, 663–670 (2003).

3. Eng, J. K., McCormack, A. L. & Yates, J. R. An approach to correlate tandem mass spectral data of peptides with amino acid sequences in a protein database. *J. Am. Soc. Mass Spectrom.* **5**, 976–989 (1994).

4. Käll, L., Canterbury, J. D., Weston, J., Noble, W. S. & MacCoss, M. J. Semi-supervised learning for peptide identification from shotgun proteomics datasets. *Nat. Methods* **4**, 923–925 (2007).

5. MacLean, B. *et al.* Skyline: an open source document editor for creating and analyzing targeted proteomics experiments. *Bioinforma. Oxf. Engl.* **26**, 966–968 (2010).

6. Gessulat, S. *et al.* Prosit: proteome-wide prediction of peptide tandem mass spectra by deep learning. *Nat. Methods* **16**, 509–518 (2019).
