# Supplementary material for: Quantitative proteomics analysis of Angiostrongylus vasorum-induced alterations in dog serum sheds light on the pathogenesis of canine angiostrongylosis
Source: Sci Rep. 2021 Jan 11;11:283. doi: 10.1038/s41598-020-79459-9 (PMC7801463; doi:10.1038/s41598-020-79459-9)
Supplement: Supplementary file 4 — Supplementary Information 4. [file 41598_2020_79459_MOESM4_ESM.docx]

**Quantitative proteomics analysis of *Angiostrongylus vasorum*-induced alterations in dog serum sheds light on the pathogenesis of canine angiostrongylosis**

Lucienne Tritten, Nina Gillis-Germitsch, Tobias Kockmann, Manuela Schnyder


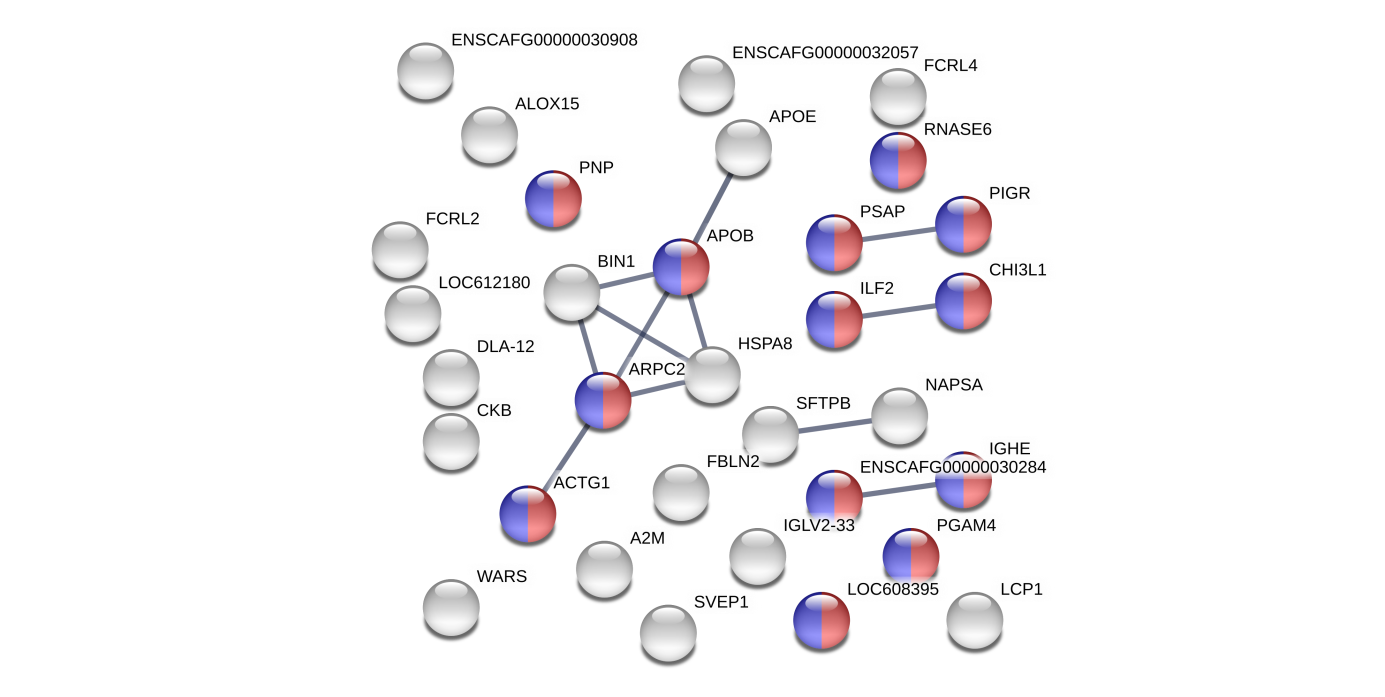


**Fig. S1. Functional protein-protein interactions among upregulated proteins at day 75 compared to day -7.** Red: Reactome pathway “innate immune sytem” (CFA-168249: FDR = 3.09e-08), Blue: Reactome pathway “immune system” (CFA-168256, FDR = 1.32e-05). A2M: alpha 2-macroglobulin, ACTG1: actin, gamma 1, ALOX15: arachidonate 15-lipoxygenase, APOB: apolipoprotein B , APOE: apolipoprotein E, ARPC2: Arp2/3 complex 34 kDa subunit, BIN1: bridging integrator 1, CHI3L1: chitinase 3-like 1, CKB: creatine kinase B-type, DLA-12: DLA class I histocompatibility antigen, A9/A9 alpha chain, ENSCAFG00000030284: uncharacterized protein, ENSCAFG00000030908: uncharacterized protein, ENSCAFG00000032057: uncharacterized protein, FBLN2: fibulin 2, FCRL2: Fc receptor-like 2, FCRL4: Fc receptor-like 4, HSPA8: heat shock protein 70 family, IGHE: immunoglobulin heavy constant epsilon, IGLV2-33: immunoglobulin lambda variable 2-33, ILF2: interleukin enhancer binding factor 2, LCP1: lymphocyte cytosolic protein 1, LOC608395: uncharacterized protein, LOC612180: uncharacterized protein, NAPSA: napsin A aspartic peptidase, PGAM4: phosphoglycerate mutase family member 4, PIGR: polymeric immunoglobulin receptor, PNP: purine nucleoside phosphorylase, PSAP: prosaposin, RNASE6: ribonuclease A family member k6, SFTPB: surfactant protein B, SVEP1: sushi, von Willebrand factor type A, EGF and pentraxin domain containing 1, WARS: tryptophanyl-tRNA synthetase
