# Supplementary material for: Quantitative proteomics analysis of Angiostrongylus vasorum-induced alterations in dog serum sheds light on the pathogenesis of canine angiostrongylosis
Source: Sci Rep. 2021 Jan 11;11:283. doi: 10.1038/s41598-020-79459-9 (PMC7801463; doi:10.1038/s41598-020-79459-9)
Supplement: Supplementary file 5 — Supplementary Information 5. [file 41598_2020_79459_MOESM5_ESM.docx]

**A**

WALTAPPGYR

**B**

SPGSSLDVTFR

**C**

NAEPGLFPWQALIVVEDTSR

**D**

TLSDVLQYVK

**E**

CSHEDSAGPALASLPSEWSPQESK

**F**

QACLSSWLDR

**G**

IAQYYYSFK

**H**

NGYISDVK

**I**

LIQNGYFHPIK

**Fig. S2 (A-I).** Calibration curves for stable isotope-labeled (SIL) peptides measured in digested matrix. Graphs comprise measurements of SIL peptides diluted 5-fold in 6 serial dilution steps and one blank sample in duplicates. Calibration curves were fitted in Skyline using unweighted linear regression in log transformed space (log-log model). LOD: Limit Of Detection (purple vertical line), LOQ: Limit Of Quantification (green vertical line). LOD was calculated based on blank measurement (manual integration at expected retention time) plus two standard deviations. LOQ was determined by max. 20% coefficient of variation (cv) and max. 20% bias. Note: Analyte concentration is based on expected synthesis yield according to vendor.
